# Developing a programme theory for a transdisciplinary research collaboration: Complex Urban Systems for Sustainability and Health

**DOI:** 10.12688/wellcomeopenres.16542.2

**Published:** 2021-07-14

**Authors:** Gemma Moore, Susan Michie, Jamie Anderson, Kristine Belesova, Melanie Crane, Clément Deloly, Sani Dimitroulopoulou, Hellen Gitau, Joanna Hale, Simon J. Lloyd, Blessing Mberu, Kanyiva Muindi, Yanlin Niu, Helen Pineo, Irene Pluchinotta, Aarathi Prasad, Anne Roue-Le Gall, Clive Shrubsole, Catalina Turcu, Ioanna Tsoulou, Paul Wilkinson, Ke Zhou, Nici Zimmermann, Michael Davies, David Osrin

**Affiliations:** 1 Institute of Environmental Design and Engineering, Bartlett School of Environment, Energy and Resources, University College London, London, WC1H 0NN, UK; 2 Clinical, Educational and Health Psychology, Division of Psychology and Language Sciences, University College London, London, WC1E 7HB, UK; 3 Buro Happold, Manchester, M3 4LZ, UK; 4 Centre on Climate Change and Planetary Health and Department of Public Health, Environments and Society, London School of Hygiene and Tropical Medicine, London, WC1H 9SH, UK; 5 Sydney School of Public Health, University of Sydney, Sydney, 2006, Australia; 6 Department of Environmental and occupational Health, EHESP, Rennes, 35000, France; 7 Air Quality and Public Health, Environmental Hazards and Emergencies Dept, Centre for Radiation, Chemical and Environmental Hazards, Public Health England, Chilton, OX11 0RQ, UK; 8 African Population and Health Research Center, Nairobi, Kenya; 9 Centre for Behaviour Change, University College London, London, WC1E 7HB, UK; 10 Climate and Health Programme (CLIMA), Barcelona Institute for Global Health (ISGlobal), Barcelona, 08003, Spain; 11 State Key Laboratory of Infectious Disease Prevention and Control, Collaborative Innovation Center for Diagnosis and Treatment of Infectious Diseases, National Institute for Communicable Disease Control and Prevention, Chinese Center for Disease Control and Prevention, Beijing, 102206, China; 12 Institute for Global Health, University College London, London, WC1N 1EH, UK; 13 Bartlett School of Planning, University College London, London, 1WC 0NN, UK

**Keywords:** Programme theory, programme evaluation, urban health, planning techniques, intersectoral collaboration

## Abstract

**Background: **Environmental improvement is a priority for urban sustainability and health and achieving it requires transformative change in cities. An approach to achieving such change is to bring together researchers, decision-makers, and public groups in the creation of research and use of scientific evidence.

**Methods: **This article describes the development of a programme theory for Complex Urban Systems for Sustainability and Health (CUSSH), a four-year Wellcome-funded research collaboration which aims to improve capacity to guide transformational health and environmental changes in cities.

**Results: **Drawing on ideas about complex systems, programme evaluation, and transdisciplinary learning, we describe how the programme is understood to “work” in terms of its anticipated processes and resulting changes. The programme theory describes a chain of outputs that ultimately leads to improvement in city sustainability and health (described in an ‘action model’), and the kinds of changes that we expect CUSSH should lead to in people, processes, policies, practices, and research (described in a ‘change model’).

**Conclusions: **Our paper adds to a growing body of research on the process of developing a comprehensive understanding of a transdisciplinary, multiagency, multi-context programme. The programme theory was developed collaboratively over two years. It involved a participatory process to ensure that a broad range of perspectives were included, to contribute to shared understanding across a multidisciplinary team. Examining our approach allowed an appreciation of the benefits and challenges of developing a programme theory for a complex, transdisciplinary research collaboration. Benefits included the development of teamworking and shared understanding and the use of programme theory in guiding evaluation. Challenges included changing membership within a large group, reaching agreement on what the theory would be ‘about’, and the inherent unpredictability of complex initiatives.

## Introduction

We describe the development of a programme theory for Complex Urban Systems for Sustainability and Health (CUSSH), a four-year Wellcome-funded research collaboration between six cities on three continents and 13 institutions. The aim of the programme is to stimulate city transformation for improved environmental quality, sustainability, and health by bringing together groups of researchers, decision-makers such as policymakers, public health, and built environment professionals, and public groups in the development and use of research evidence. International multi-partner transdisciplinary programmes are increasingly desired and supported by research funders and government bodies, but there is a need to understand ‘what works’ in practice. Despite the interest in such programmes, difficulties can arise when researchers from different disciplines work with each other and with partners from other sectors ^1, 2^.

### Transdisciplinary research

Transdisciplinary research is a challenging undertaking. Defined as *‘*an integrative process whereby scholars and practitioners from both academic disciplines and non-academic fields work jointly to develop and use novel conceptual and methodological approaches that synthesize and extend discipline-specific perspectives, theories, methods, and translational strategies to yield innovative solutions to particular scientific and societal problems’ ^3^, it requires individuals with diverse backgrounds to draw on different sources and forms of knowledge at scales from local to global ^4^. Multiple partners need to interact in different capacities and at different levels to generate and use findings within complex social and political systems and sometimes in different geographical contexts ^5^. The development of a programme theory was an opportunity to examine how a cross-cultural, geographically dispersed collaboration with many moving parts might ‘work’ ^6^, both in achieving its aim of promoting city health and sustainability, and in understanding the processes and outcomes of transdisciplinary research.

### Programme theory

Programme theory describes a variety of ways of developing a model that links programme inputs and activities to a chain of intended outputs and observed outcomes, and then uses the model to guide evaluation ^7, 8^. This kind of theory-based evaluation often rests on logic models or theories of change ^9^. Complex interventions can be difficult to evaluate because they are based on poorly articulated assumptions ^6^. A programme theory, therefore, seeks to open the “black box” between inputs and outputs ^10^. Complex programmes—with multiple components, uncertain pathways, and emergent outcomes—present a challenge to the development of programme theories and subsequent evaluation. Their structure often includes several layers working simultaneously: individuals, communities, local, regional, and national government. It includes a range of perspectives, involves variability in delivery processes, and is of broad scope in real-world settings. If, however, we want to understand the success of such programmes and how they can be constructed, we need to understand their mechanics. Developing a programme theory can help investigate and evaluate the implementation process and the means by which change is brought about ^11^. Despite the challenges, numerous examples in practice illustrate the possibilities of developing useful programme theories, although often from a single disciplinary or geographical perspective ^12– 15^. This paper contributes to the body of knowledge on developing programme theories, providing a detailed account of the process of development as an example for a complex, transdisciplinary programme.

### Theory development as a process

Our work offers a rationale for developing a programme theory through collaborative and participatory processes and stresses its role as a provisional framework to guide evaluation. Developing a programme theory for CUSSH was important as a means of generating consensus between partners on the ways in which programme success might be evaluated. It required discussion of core assumptions, beliefs about the relationships between research and policymaking, and ideas about the kinds of indicators that might be used to measure progress and define “success” ^16^. These discussions and the iterations of the programme theory can contribute to a process of team building, examining preconceptions and achieving a shared language. A collaborative process involving contributions from different forms of knowledge can help understand conceptual connections across the work of different partners, thereby guiding the corresponding collaboration with a purpose aligned with the agreed objectives. The clarifications that result from the process and the visual presentation of the theory contribute to a framework for programme evaluation ^7– 9^: the success of implementation (whether theoretical steps happened in practice) and whether the programme worked as expected (whether theoretical assumptions about how the activities would lead to change actually held) ^17– 22^. A programme theory and evaluation framework would enable the CUSSH team to compare experience with expectation and better understand the processes necessary to drive changes in health and environmental sustainability ^23^.

For some programmes, a programme theory is developed and finalised at the start, although this is difficult in a complex programme in which new learnings are brought together. In practice, the development of a complex programme takes place as an iterative process. In this paper, we summarise the process and product, beginning with a sketch of the programme and then describing the development and content of its theory. We include some examples from our work in partner cities to illustrate the links between theory and practice. We hope that the paper contributes to developing knowledge about how to construct and evaluate a complex, transdisciplinary research programme.

### The CUSSH programme

The programme’s aim is to enable city decision-makers to select and implement optimal actions for both sustainability and health. The objectives are to provide research evidence on the environmental and health benefits of large-scale city initiatives, to enable and support cities to deliver significant, measurable change by engaging and collaborating with stakeholders, and to find solutions that work in complex systems by fostering a systems thinking approach. The programme will also test the degree to which the use of scientific evidence and participatory engagement in decision processes can strengthen the envisioning, design, and implementation of transformational health and sustainability policies.

These objectives and aspirations are addressed in five broad, interacting work packages:

1. Examining existing evidence on the relative effectiveness of potential city actions.
2. Developing methods to track progress toward environmental and health goals.
3. Using modelling and microsimulation to develop rapid assessment tools for city actions.
4. Engaging decision-makers in partner cities in applying evidence-based systems thinking to help in policy and planning.
5. Engaging citizens in the decision-making process.

Central to the collaboration is partnership with six cities with different socio-political, geographical, environmental and city size contexts: Nairobi and Kisumu in Kenya, Beijing and Ningbo in China, and Rennes and London in Europe. Each geographical pair of cities includes a capital and a smaller city. The aim was to develop a matrix of contrasting income levels, environmental challenges, and scale, within pragmatic and logistical boundaries. The choice of capitals arose primarily as locations of academic research partners: Nairobi for the African Population and Health Research Centre, Beijing for Peking University, and London for University College London and the London School of Hygiene and Tropical Medicine. The choice of smaller cities arose from existing connections: with Kisumu County for its interest in environmental initiatives to address its challenges of expansion, Ningbo, recommended because of its rapid expansion, and Rennes for its municipal commitment to urban health and sustainability.

City environments depend on human activity modifying the natural environment to create, connect and sustain systems for living and working. They are made up of two sorts of fabric: the “hard” fabric of buildings, infrastructure, and bounded open space, and the “soft” fabric of community, society, and governance (the *ville* and the *cité*) ^24^. Because achieving sustainability and health will both depend on changes to the built and social environments, the collaboration includes experts on hard fabric (engineering, building physics), soft fabric (urban sociology, behavioural science), public policy and decision-making (urban planning and public health), environment and health (environmental science, climate science, urban health, planetary health), and methodologies (modelling and simulation, participatory system dynamics, public engagement, programme evaluation, evidence synthesis).

The partnership agreed on three foundational ideas from the beginning: that transformative change in cities is necessary, that research evidence should inform policymaking and planning for change, and that cities are complex and require transdisciplinary solutions.

### Transformative change in cities is necessary

Environmental improvement is a priority for both sustainability and health and actions to improve either have the potential to provide co-benefits and improve both ^25– 29^. However, city-level efforts at the scale necessary to meet sustainability goals or improve population health have been inadequate ^30– 32^. Actions have involved largely transitional changes, or piecemeal actions, which may be useful in developing capacity for change—in harnessing individuals, organisations, and society to develop and adapt—but are unlikely to be at a sufficient scale and pace to meet current imperatives. Radical and rapid shifts in infrastructural, behavioural, and operational systems are required to transform cities to meet health and sustainability needs. Table 1 summarises some general comparisons between urban change that we would define as ‘transitional’ and the type of urban transformation required to meet health and sustainability goals.

**Table 1.** Conceptualising and comparing transitional and transformational city change.

CHARACTERISTICS OF CHANGE	TRANSITIONAL	TRANSFORMATIONAL	REFERENCES
**Semantics**	Quantitative change “Change IN city”	Qualitative change “Change OF city”	34
SCALE	Small Sub-city level	Large City level	31, 34,
**Type**	Grassroots Niche innovations	Multi-dimensional Change in structures, environments, practices, culture	35, 36
**Timeframe**	Long-term process of diffusion	Immediate	37
PURPOSE	Incremental	Inadvertent or purposive	38
**Perspective**	Localised Can be isolated	Systemic	36, 39, 40

### Research evidence should inform policymaking and planning

Policymaking is non-linear and scientific evidence–even if cited—often contributes little to the decision-making process ^33^. Although research can inform and add weight to recommendations ^41^, it has not often been used to inform urban policy directly ^42, 43^. Academics have begun to address this challenge through engagements with multiple stakeholders that examine trade-offs, feasibility, and effects in the short and long term ^44^. These engagements aim to increase communication between researchers and policymakers working on a range of time-sensitive projects and programmes ^45, 46^, using evidence to respond to local concerns, address a salient issue at the right time ^47, 48^, and tell a persuasive and relatable story with people’s lives at its centre ^43, 49^. In a systematic review of barriers to and facilitators of policymakers’ use of evidence ^1^, the most frequent needs were to increase the dissemination and availability of research findings, to make them as clear and relevant as possible (particularly to users with limited experience of research methods and their interpretation), and to encourage collaboration between policymakers and researchers. A key theme is how academics build relationships with policymakers. A recent review by Oliver and Cairney notes that academics have strong incentives to influence policymaking, but may not know where to start ^50^.

### Cities are complex and require transdisciplinary engagement and a spectrum of methods

Cities provide examples of the ‘wicked’ problems characteristic of complex systems ^51, 52^. Understanding change in cities requires different disciplines to work together, incorporating a spectrum of methods, systems thinking, and participatory engagement ^43, 53– 55^. Systems thinking is about incorporating a variety of ways of thinking and views about a problem or challenge to generate a more comprehensive understanding of cause-and-effect ^56^. A complex view of cities means seeing parts of the system holistically as a set of elements whose interactions may affect the whole more than the parts, with properties emerging at different levels, causality operating both within and between levels, and people acting according to different rationales ^57^. We took a structured approach to addressing sustainability and health in cities: clarifying the issues that need to be addressed, investigating their causes, developing solutions, and supporting implementation. The systems thinking approach helped us understand feedback systems, local networks, infrastructure, decision-making processes, and power relations. In a related effort to break the cycle of risk accumulation across sub-Saharan African cities, Dodman and colleagues provide a rationale for using a spectrum of methods to address a spectrum of risks, demonstrating in a wide-ranging multi-country programme of research the utility of mixed-methods approaches in planning for resilience ^58^. They galvanised multidisciplinary expertise and multifaceted and collaborative research to gather empirical data and build a strong evidence base covering social, political, economic, biophysical, and hydrogeological systems in cities to provide a more solid base for planning and investment.

The need to work with such complex biosocial and technical systems has led to calls for transdisciplinarity ^28, 59– 63^, encapsulated in ideas about the Science of Team Science ^64, 65^. Generally, working with cities is an attractive proposition because their political structures and decision-making powers can respond flexibly to challenges (city mayors, for example, have become prominent on the global stage), but achieving change will involve both city decision-makers and planners and other actors—formal and informal—who affect implementation. Table 2 summarises the different stakeholder groups involved.

**Table 2.** Complex Urban Systems for Sustainability and Health (CUSSH) stakeholders: what do we mean by policymakers, decision-makers, planners, policy entrepreneurs, governance, and civil society actors?

Term	Definition	Partners in CUSSH
**Policymakers**	Public policy can be defined as ‘the deliberate decisions - actions and nonactions - of a government or an equivalent authority toward specific objectives’ ^75^. Of the many actors involved in setting public policy agendas, those affiliated with government who are taking policy decisions are considered policymakers.	Policymakers working with CUSSH include national and local government actors working within health, environment, housing, and other sectors.
**Decision-** **makers**	Decision-makers is a broad term covering a range of stakeholders. Within public policy, the actors taking decisions may be affiliated with government or acting on its behalf.	Decision-makers working with CUSSH include a range of stakeholders who influence the urban environment: national and local governmental staff, planners, developers, financiers.
**Planners**	Urban planning is the ‘well-honed practice of systematic compromise’ ^76^, or the process of moving from knowledge to action ^77^. Planning’s purpose and means are contested and diverse ^78^. Planners shape and apply regulations about land use, take decisions in the interest of the public, balance competing interests, and seek to increase transparency and participation in local decision-making.	Within some of the CUSSH cities urban and city planners are involved in the programme.
**Policy** **entrepreneurs**	Actors who use their power, knowledge, and networks to achieve their goals by influencing policy. They may be consultants, non- governmental organisations, politicians, lobbyists, or others ^79^.	Policy entrepreneurs working with CUSSH include non-government organizations working within the health, environment, and sustainability sectors.
**Civil society** **actors**	Actors involved in public policy include ‘individuals or collectives such as organizations, networks, or coalitions—and their attributes, including their knowledge, values, beliefs, interests, strategies, and resources’ ^75^.	Civil society actors working with CUSSH include public and community groups and networks within the cities.

## Methods

The process of co-developing and agreeing the programme theory has been contentious and time-consuming, but ultimately valuable. In summarising our methods, we distinguish between two strands of work: developing the programme theory itself and developing knowledge that contributed to it.

### Developing the programme theory

The current version was developed over two years from June 2018 to August 2020, through discussions of a series of 18 versions, each followed by individual and collective feedback. Table 3 summarises the sequence of activities. Retrospectively, there were four loose stages to the participatory process. In the first stage, partners were introduced to the idea of programme theory and considered the themes of the collaboration. In the second stage, these themes were framed in a series of drafts of the theory. The third stage considered the theory as a framework for evaluation, and the fourth stage finalized the programme theory and supporting documentation.

**Table 3.** Stages of Complex Urban Systems for Sustainability and Health (CUSSH) programme theory development.

Stage	Action	Year	Month	Number of CUSSH members involved	Approach to recruitment Meeting format
1. Generating ideas on the theory	Small group discussion and meeting about programme theory	2018	June	6	Sub-group of CUSSH team identified with skills and interest in developing the programmetheory Open discussion
	Agenda item at all team meeting		June	11	Open invitation to whole CUSSH team Agenda item alongside CUSSH updates
	Workshop on transformative change		September	17	Open invitation to whole CUSSH team Workshop organised by members of CUSSH team (work package 1)
	Agenda item at all team meeting	2019	April	10	Open invitation to whole CUSSH team Agenda item alongside CUSSH updates
	Session at annual meeting		May	25	Open invitation to whole CUSSH team Several external stakeholders Presentations and discussion
	Agenda item at team meeting		June	9	Open invitation to whole CUSSH team Agenda item alongside CUSSH updates
2. Sharing ideas	Circulation of draft paper and documents		April	16	Sub-group of CUSSH team identified with skills and interest in developing the programme theory Open discussion
	Workshop on transformative change		May	20	Open invitation to whole CUSSH team Workshop organised by members of CUSSH team (work package 1)
	Small group discussion and meeting		July	16	Sub-group of CUSSH team identified with skills and interest in developing the programme theory Email notes and revisions
	Agenda item and discussion at all team meeting		October	8	Open invitation to whole CUSSH team Agenda item alongside CUSSH updates
3. Reflecting on the theory	Small group discussion and meeting		November	16	Sub-group of CUSSH team identified with skills and interest in developing the programme theory Email notes and revisions
	Involvement of team member focused on evaluation	2020	January	1	Independent review of documents
	Senior management team meeting to discuss programme theory		January	8	Senior management team of CUSSH Agenda item alongside CUSSH updates
4. Finalising the theory and supporting documentation	Circulation of key documents to senior management team		May	5	Senior management team of CUSSH Email notes and revisions
	Session at annual meeting		June	55	Open invitation to whole CUSSH team Several external stakeholders Presentations and discussion
	Targeted involvement of specific members of CUSSH in follow-up discussions and drafting		June	8	Targeted requests sent to members of CUSSH team Review and editing of documents
	Evaluation working group established		July	15	- Sub-group of CUSSH team identified with skills and interest in developing the programme theory Open discussion and follow up emails
	Circulation of draft paper to members of the team		July	25	Members of CUSSH who have been involved in or have interest in programme theory Draft paper and diagrams with comments

***Stage 1: Generating ideas about theory (~12 months)***

In the first year of the collaboration, discussions encouraged collective understanding of the rationale for programme activities. The diversity of the partnership meant that individual and institutional objectives and assumptions about how objectives could be met differed and were often tacit rather than explicit. The process of developing the programme theory began with a series of meetings with CUSSH collaborators, including researchers, decision-makers, and non-government advocates. It was followed by two similar meetings of about 15 people which considered approaches to developing and specifying programme theories ^9, 17, 18, 66– 68^, and theories of change ^6, 69, 70^. These discussions led to two pieces of work. The first was a review of the global literature on transforming cities for sustainability and health, along with consideration of existing frameworks for understanding urban health and environmental sustainability ^27, 30, 51, 71, 72^. The second was a logic model developed from the original research proposal and reports of case studies from the six partner cities ( Table 4). It represented potential pathways to change, organised in terms of inputs, mediators, outcomes, and impact and informed by the Medical Research Council guidelines for complex interventions and for process evaluations ^11, 73, 74^.

**Table 4.** Complex Urban Systems for Sustainability and Health (CUSSH) logic model developed in initial discussions.

Background information	CUSSH Inputs (these interact)	Mediators (mapped against inputs)	Outputs	Impact
• City governance structure, geographic, and population characteristics • City policies (especially those that support change in cities), plans (city documents) • Past, present and projected data (city datasets and reported statistics) • Informal evidence about current situation, potential levers and barriers (written and verbal communication) • Capabilities, opportunities and motivation (including goals, incentives, values) of stakeholders ○ Organisations and individuals ○ Who to engage, for what, how best to engage	• Scientific evidence (global, national & local) • Impact & system dynamic models • City reports • Capacity development • Expertise: policy & data analysis; policy/ intervention design including analysis of unintended consequences and trade-offs of policy options; behavioural analysis (including identifying target behaviours, levers and barriers); implementation design and intervention; application of theories of change; • Evaluation methods • CUSSH human resources • Participatory and solution- focused workshops • Public engagement activities • Support for funding bids • Partnership, engagement and co-design • Capacity development • Securing additional funding	For key stakeholders, INCREASING **CAPABILITY** (knowledge and skills) INCREASING **OPPORTUNITY** (social and material) INCREASING **MOTIVATION** (incl. beliefs, attitudes, emotions)	For cities: • Actionable policies • Implementation strategies – who, what, when, where, how • Project reports For science: • Scientific articles • Conference presentations • New collaborations • Cumulative research agenda	For cities: • Implemented policies amounting or leading to incremental or transformative change • Increased capacity to enable future change in our partner cities and trigger or support similar processes in other cities For Science: • Advanced understanding of how different disciplines can work together to bring about transformative change • Advance understanding of how to bring about city-wide transformative change, • Lessons learnt about what methods were not effective, and why

The literature review and logic model informed two workshops in September 2018 and May 2019, with participants from all partner countries at an annual CUSSH meeting bringing together a diversity of perspectives. Participants included academics from a range of disciplines, city partners, and those responsible for programme implementation in cities. The first workshop captured responses to the work to date and contributed ideas for theory development. The second summarised participants’ ideas about program theory in general and discussed approaches to theory development. At both workshops, participants worked in small groups to generate ideas ^16, 19, 80– 83^.

***Stage 2: Sharing ideas (~6 months)***

There followed regular meetings of members of the broader study team who had expressed interest in this piece of work, developing the programme theory in documented iterations. After seven meetings and 14 versions, the theory was circulated to the wider partnership for comments. The early versions focused on the details of one programme theory diagram.

***Stage 3: Reflecting on the theory (~6 months)***

Up to this point, the programme theory was a broad conceptual map for CUSSH, both for exploring the general context of the programme and for strategic planning related to its delivery in specific cities. The third stage involved breaking down, rebuilding, and reframing the theory as a framework for evaluation. An evaluation specialist informally interviewed team and partnership members to understand their ideas about the programme theory and evaluation. Subsequent discussions, recirculation, and adaptation led to the 18 ^th^ and current version. The programme theory became an ‘action model’ and ‘change model’ (outlined below).

***Stage 4: “Finalising” the theory***

The programme theory was shared across the CUSSH collaboration in preparation for an annual meeting in June 2020. It was used as a way of structuring discussions at the meeting: a session was devoted to it and the remaining sessions were framed around its cogency and completeness. At this point the theory moved from a relatively passive to an active role in the delivery of the programme. The current version of the theory draws on recent work on transdisciplinarity (Pineo *et al*., Health Promotion Int 2021, in press).

### Developing knowledge that contributed to the programme theory

Here, we summarise the activities in partner cities that informed our discussions of programme theory, but for which theory development was not the object. These experiences informed the content of Box 1– Box 4 later in the article. Details of methods will be available in publications that result directly from the work.

**Box 1. Protracted timescales for building relationships and consensus: France**

In Rennes, several factors helped to build relationships and consensus between academic partners and city stakeholders. Nevertheless, the development phase of the transdisciplinary collaboration took more than a year to progress. The integration into Complex Urban Systems for Sustainability and Health (CUSSH) of a local scientific team from the School of Public Health (EHESP), a year after the programme began, was a facilitating factor. Thanks to existing collaboration, notably through the Réseau Bretagne Urbanisme et Santé (RBUS) urban planning network that had existed for ten years, EHESP and city stakeholders were able to accelerate the exchange between CUSSH and local decision-makers. For active and sustainable involvement of city actors in urban planning, environmental, and public health, it was decided to co-draft a "scope of work". This process allowed partners to exchange and align their expectations. Although it took time, it meant that the concerns of practitioners and decision-makers would be better understood and addressed in the project and that they would, in turn, be more attentive to the findings of CUSSH research. The scope of work was annexed to the scientific collaboration agreement and to a Memorandum of Understanding signed by all stakeholders. Among the internal governance structures set up to maintain the collaboration were fortnightly meetings with partners from the city of Rennes urban planning department and the public health department, and the addition of CUSSH-specific items to the agenda of quarterly RBUS network meetings.

**Box 2. Challenges to building relationships and consensus: Kenya**

In Kenya, public engagement workshops were held to come to an agreement on the areas of focus for Kisumu and Nairobi. The workshops ensured that residents contributed to the discussions of what they felt would be priority areas for the cities to focus on. The workshops raised the issue that project implementation might face resistance to change from groups with vested interests. For some it was believed that cartels influenced sectors such as transport, solid waste management, lands, and water. For example, cartels in the solid waste management sector in Nairobi demanded ‘security’ levies from vehicles entering dumpsites ^84^. This may lead to illegal dumping of waste in the city as service providers avoid municipal dumpsites, for which they already had to pay. The current leadership of Nairobi Metropolitan Services have promised to explore the issue and disband the cartels for better service delivery to residents.

Political processes such as campaigns and elections may indirectly influence the programme’s implementation. County leaders are usually elected into office, after which they appoint heads of departments, most often with political affiliation. The heads of departments lead teams of professionals, and this is usually where divergence of views begins. While the leadership may consider the political influence, professionals are expected to deliver in their fields of expertise. The Complex Urban Systems for Sustainability and Health (CUSSH) programme experienced a delay in engaging with Nairobi County because of frequent changes in leadership, which meant that new relationships had to be established. For example, the creation of the Nairobi Metropolitan Services brought in new leaders from whom we had to seek commitment for CUSSH. Meeting them has not been easy given that their priorities and performance targets differ from those of the partnership. Bureaucracy in decision-making and financial constraints within governing bodies have also contributed to delays.

**Box 3. Understanding city context: United Kingdom**

In London, stakeholders in a Thamesmead case study have been involved in a participatory System Dynamics modelling process involving workshops and interviews. A shared concern and the modelling objectives were defined jointly and maps such as Causal Loop Diagrams (CLDs) used to elicit the knowledge of individuals and teams. CLDs are commonly adopted to communicate important feedback on a problem within a decision-making process, capturing a variety of information. After building and comparing the different CLDs, participants discussed the main unexpected and known dynamics. Based on similarities and differences identified in the perceptions of system boundaries by different stakeholder groups, stakeholders discussed what changes they would like to generate in Thamesmead.

**Box 4. Synthesising city evidence: China**

The Chinese Center for Disease Control and Prevention (China CDC) delivers China’s plan and guidance for disease prevention and control and public health, under the direction of the National Health Commission of the People’s Republic of China. China CDC is the key Complex Urban Systems for Sustainability and Health (CUSSH) partner in co-designing and implementing city case studies and projects in Beijing and Ningbo. Early engagement with China CDC allowed the partners to identify shared research interests and practical roles. Communication between the partners made it possible to understand the broad context of China and how CUSSH could engage with decision-making. A range of research topics were included in case studies, including the use of evidence across stakeholders, decision-making in building sustainable and healthy cities in China, and specific environmental challenges such as air pollution. Interviews with stakeholders from academia, government research agencies, and other organisations in China suggested a systems thinking approach for control of air pollution. This clarified the drivers of decision-making on environmental and health issues and provided rich information on Chinese city contexts.

***Beijing and Ningbo.*** The Chinese Center for Disease Control and Prevention (China CDC), which delivers China’s plan and guidance for disease prevention and control and public health, under the direction of the National Health Commission of the People’s Republic of China, is the key CUSSH partner in co-designing and implementing city case studies and projects in Beijing and Ningbo. Early engagement with China CDC allowed the partners to identify shared research interests and practical roles. Communication between the partners made it possible to understand the broad context of China and how CUSSH could engage with decision-making. Work relevant to the programme theory drew on analyses of a series of semi-structured qualitative interviews with key informants in May 2019 (publication in preparation). We interviewed 12 participants in Beijing and 11 in Ningbo, purposively sampled based on their professional networks to include people with knowledge of urban sustainability and health challenges and associated policy processes. Participants were identified and invited by partners at China CDC. They came from municipal research and administration agencies, university departments, community service centres, and primary care services. The interview guide was informed by a pre-identified conceptual framework and agreed through discussion with the wider research team. Translators were included in the interviews, which took place at China CDC offices in Beijing and Ningbo or participants’ offices. Participants had professional expertise in climate change, water, soil and air pollution, medicine, public health and epidemiology, urban planning, economic development, meteorology, and waste management. Interviews covered the use of evidence across stakeholders, decision-making in building sustainable and healthy cities in China, and specific environmental challenges such as air pollution.

***London.*** Work in London relevant to the programme theory drew on a case study of a participatory system dynamics modelling process in Thamesmead developed from November 2019 to July 2020. Detailed information is available elsewhere ^85^. The objectives were to bring together institutional stakeholders to jointly scope the focus of work on green and blue spaces, sustainability, and health, to build causal maps (Causal Loop Diagrams) around shared concerns to capture the system boundaries for each group of stakeholders, to understand differences in stakeholders’ perceptions of system boundaries and their potential effects on decision-making, to bring residents’ vision into the discussion, and to collectively agree on the focus of a system dynamics model. In the early stages, CUSSH researchers met with major stakeholders including the CLEVER Cities research project ( https://clevercities.eu) and Peabody housing association ( https://www.peabody.org.uk). As the direction of the case study developed, further groups were either suggested by these stakeholders or identified by CUSSH researchers as important to the context. Additional stakeholders were invited to take part in interviews or workshops. A stakeholder list was maintained and updated iteratively as new organisations or individual contacts were identified. Initial problem-scoping interviews were guided by a script to elicit views on key concerns for Thamesmead in relation to green and blue spaces, sustainability and health. These views provided a starting point for a problem-scoping workshop, facilitated by CUSSH and guided by a workshop script. Subsequent workshops were scripted to elicit further detail and gradually build an understanding of the agreed focus problem.

***Rennes.*** The development of the research collaboration in Rennes took place in several stages. Partnership development began in 2017 with a meeting between the Rennes Deputy Mayor for Health and CUSSH researchers. A series of meetings and workshops involved stakeholders from both academic and non-academic sectors. Tracking since the launch of the project identifies 18 key exchange times, including CUSSH workshops, CUSSH annual meetings, and specific meetings involving stakeholders including Réseau Bretagne Urbanisme et Santé (RBUS) and participants from the École des Hautes Études en Santé Publique (EHESP). These exchanges led to the elaboration of a Scope of Work, implementation of an evaluation tool, and installation of an internal governance system that has consolidated the research collaboration. A feature of the collaboration is the involvement of actors from the City of Rennes and Rennes Metropole in the research process. Participants include point contacts for the City of Rennes, a project manager from the urban planning department of Rennes Métropole, the Director of the Health and Environment Department of the City of Rennes, along with stakeholders such as elected decision-makers and city managers, the EHESP scientific team, Buro Happold, and CUSSH management and scientific teams.

***Nairobi and Kisumu.*** In the Kenyan cities, partnerships with both county governments were built during the pilot phase under the Housing in Nairobi’s Informal Settlements programme (HINIS), which ran from 2016 to 2017 and sought to test participatory approaches. While HINIS focused on Nairobi, we invited Kisumu county officials to attend the workshops. When CUSSH was launched, we built on these relationships, met with the governor of Kisumu county to brief him, and met a cross-section of local government representatives to discuss the project. For each city, we selected a liaison officer who would be the face of CUSSH in the city government, provide updates to the relevant departments on CUSSH activities, and provide city-level information needed by the CUSSH team.

Work relevant to the programme theory drew primarily on workshops in Kisumu. Participants were identified and invited by the county government liaison officer. The first workshops in July 2018 were broad and aimed to discuss the challenges the city faces and agree on the issues that the CUSSH programme would work on. Participants included representatives of the County Government, non-government and community-based organisations (NGOs and CBOs), academics working in Kisumu, and influential individuals. County officials and organisational representatives met with the CUSSH team to discuss ongoing projects within the city as well as potential collaboration to secure further funding to support either ongoing or new initiatives aimed at improving the health and wellbeing of residents. Subsequent meetings refined the focus of a concept note for submission to the Green Climate Fund. Participants in these meetings were county officials from the environment department with a focus on conversion of waste to energy.

Further activities undertaken in July 2019 included workshops, focus group discussions, and interviews with key informants. Arranged in two consecutive sessions, the workshops involved a variety of participants to agree on a local challenge to be addressed in a funding application. The challenge agreed upon was municipal solid waste management, on which subsequent fieldwork focused. Participants represented different sectors involved in local waste management, including civil servants in the county government, academics, industry and trading associations, CBOs and NGOs, and local residents. Purposive sampling was used to invite participants based on their knowledge of waste management. Participants were gathered in groups according to their sector (except for the first workshop which covered multiple sectors). Local government was represented by civil servants from departments addressing topics overlapping with waste management, including environment, climate change, energy and urban development. Invited academics had knowledge on waste management through teaching or research. A further group of participant mobilizers were individuals from CBOs or NGOs who resided in Kisumu. A participant from the sugarcane industry – a main industry for the local economy—and two from trading associations attended the focus groups. Residents’ association representatives from underserved areas were also invited.

### Ethical considerations

Low-risk ethical approval for stakeholder interviews and workshops across the programme was granted by the UCL Bartlett School of the Environment, Energy and Resources on 23 January 2018 and renewed on 18 June 2020 during the Covid-19 pandemic. In China, adjunctive approval was provided in 2019 by the China CDC. In Kenya, adjunctive approval was provided in 2018 by Amref Health Africa (P506-2018), with a subsequent research permit from the National Commission for Science, Technology and Innovation (NACOSTI/P/21/8252). Participants in all workshops and interviews gave written informed consent.

## Results

**Figure 1.**
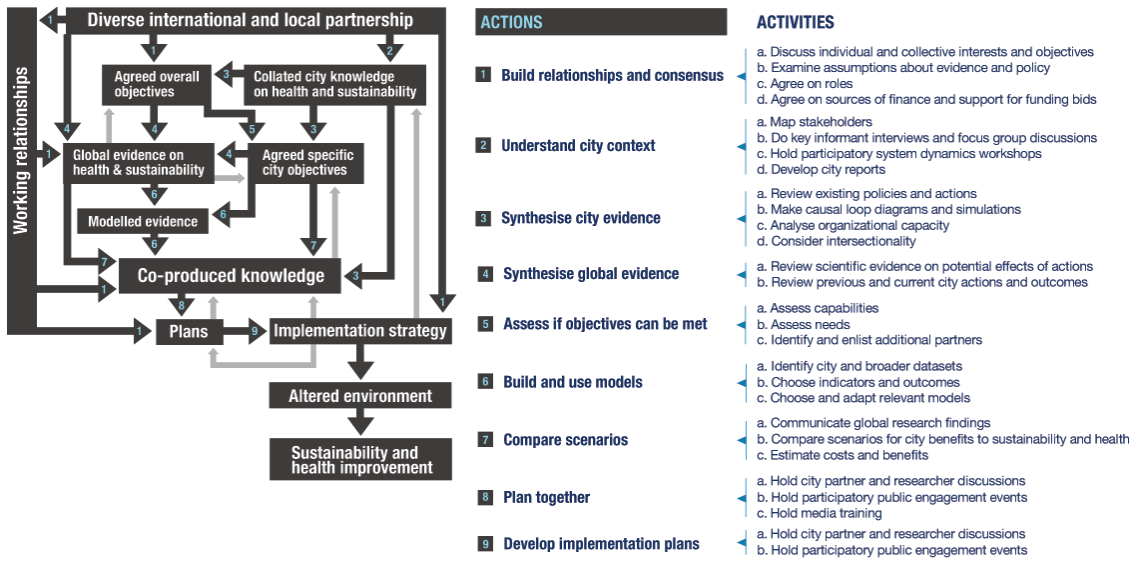
Action model for the CUSSH collaboration: change in cities. The boxes in Figure 1 represent outputs (‘nouns’: things that are produced and can be documented and measured) and the arrows represent the transition between them (‘verbs’: actions that influence movement from one box to the next). Each transition arrow on the diagram is mapped to a numbered action and activities which we expect will help achieve the transition, and which form a loose sequence described in the sections below. The overall pathway from a diverse partnership (top of diagram) to environmental and health improvement (bottom of diagram) includes a sequence of transitions over time. These transitions involve different actions and can occur simultaneously. The process is non-linear and, as befits complexity, there are feedback loops at all levels, the most obvious of which are outlined in the figure.

**Figure 2.**
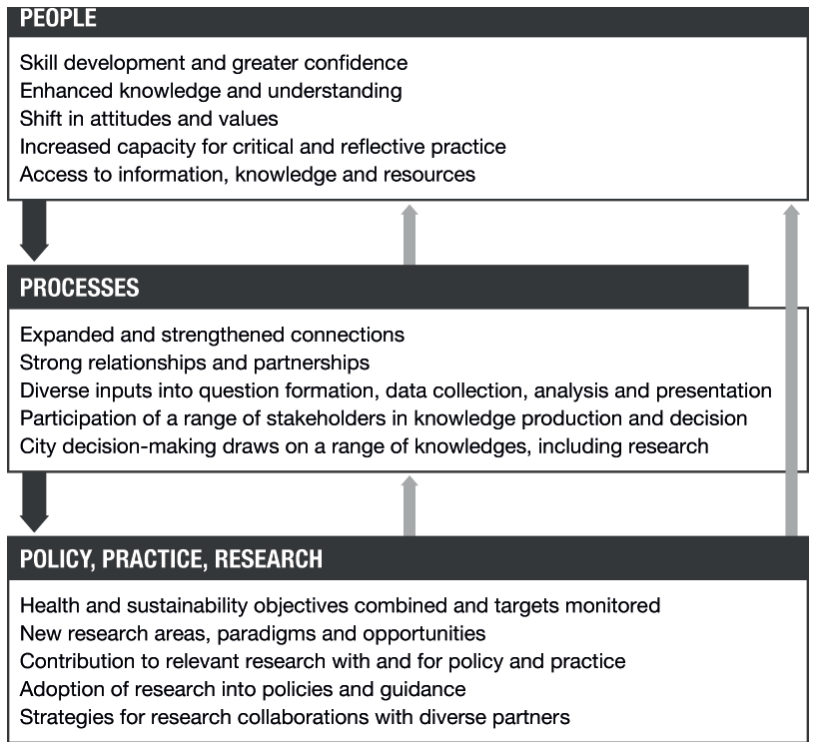
Change model for the Complex Urban Systems for Sustainability and Health (CUSSH) collaboration.

This section summarises the programme theory and provides brief illustrations from practice. Figure 1 and Figure 2 present the theory schematically and show that it addresses two different questions. The first is how the CUSSH programme helps cities to achieve health and sustainability. This is addressed by an ‘action model’ ( Figure 1). The second question is what changes result from the programme, and what has led to these changes. This is addressed by a ‘change model’ ( Figure 2). The various versions of the programme theory are available as underlying data ^86^.

### Action model

The action model describes planned activities that we expect to generate change. Co-produced knowledge is a centrally important output (middle of diagram). All of the numbered activities that culminate in co-produced knowledge and an implementation strategy can be mapped to three kinds of knowledge co-production that we identified to be important: ‘target knowledge’ or ‘where we want to get’; this requires support for participation by a range of decision-makers and citizens (setting questions, using data, disseminating findings); ‘transformation knowledge’ or ‘how to get there’; this requires bringing together knowledge from a range of disciplines within and beyond academia; and ‘systems knowledge’ or ‘how it works’; this involves drawing on systems thinking and behaviour change theory.

The action model can be summarised in a short narrative, which is further developed below in 10 stages:

CUSSH is an international partnership of researchers, decision-makers, and advocates who aim to use research for better city health and sustainability. Our diversity makes it important for us to work toward consensus by discussing our individual and collective interests and clarifying our assumptions and roles. We will collate and discuss global and local knowledge in order to agree on both our general objectives and specific objectives for individual cities. We will help decision-makers and citizens use this co-produced knowledge, along with mathematical models, to compare scenarios so that city plans are based on evidence. We will support the development of implementation strategies so that these participatory plans have the best chance of improving city health and sustainability.

***1. Build relationships and consensus***

A first step in the model, and an implicit aim of the CUSSH way of working, is the formation of a diverse partnership across countries and institutions. Working relationships can be developed through meetings and communications that discuss individual and collective interests and objectives (1a) and examine assumptions about the links between evidence and policy (1b). Partner individuals and organisations need to agree on their roles (1c) and interests. Financial arrangements must be agreed at this stage (1d), including the limits of the programme’s capacity and the kinds of activities that money will be spent on. Although this represents the development phase of transdisciplinary collaboration, roles evolve, people and organisations come and go, and the maintenance of working relationships extends throughout the programme duration, contributing to several of the transitions in Figure 2. Partners may assume that the overall objectives of the collaboration are clear, but they will vary with perspective. The frame of reference may be individual, organizational, local, citywide, national, regional, or global. There can be substantial differences in tacit assumptions and it is worth making them explicit. Box 1 and Box 2 provide practice illustrations from France and Kenya. 

***2. Understand city context***

This activity involves discussion and information gathering applied to a specific city. City knowledge on health and sustainability is co-produced through stakeholder mapping (2a), interviews (2b), participatory system dynamics workshops (2c), and synthesis of city reports (2d). The aim of this series of actions is to understand how the city works in terms of influences on decision-making. Of particular importance is an understanding of critical players who help or hinder city-level strategy ^87^, of the city’s focus of attention on certain priorities, and of the structural and financial opportunities and barriers. Box 3 provides a practice illustration from the United Kingdom.

***3. Synthesise city evidence***

This activity is local and aims to gather understanding on the contextual feasibility and potential impacts of different actions, to complement lessons learned from the academic literature. For a partner city, existing policies and plans are reviewed (3a): what has been done, what has been planned, what are the facilitators and barriers to implementation of the existing plans? Participatory systems dynamics workshops are held, in full in some settings and using a lighter framework in others to develop agreement on the issues and objectives for the city as well as the city-CUSSH collaboration (3b). Organizational attention and interests are assessed and strengths and weaknesses identified that will affect planning and implementation (3c). Intersectional inequalities are considered and demographic, sociocultural, and socioeconomic concerns elicited, so that plans may address the health of vulnerable groups and to ensure that they are represented in discussions (3d). Box 4 provides a practice illustration from China.

***4. Synthesise global evidence***

This activity involves bringing together two kinds of evidence, predominantly through literature reviews. The first is a catalogue of actions implemented in cities to benefit health and the environment, with subsequent syntheses of their implementation processes, understanding barriers, facilitators, and any unintended consequences, as well as their actual impact on health and the environment (4a). The second is an evaluation of the potential effects of actions, locally and globally: how likely they are to have a place in a menu of transformative options (4b).

***5. Assess if objectives can be met***

As objectives are agreed and city contexts are understood within the research team, gaps in particular capabilities to meet the objectives may be identified (5a), or that experienced advice may be of benefit to city partners (5b). Partners may be brought in where necessary to meet emerging capability needs (5c), which would add to the distributed partnership.

***6. Build and use models***

Models to help in participatory planning require data. There is a need to find and ensure access to datasets at multiple spatial scales (6a). In order to debate the potential effects of city actions, the indicators upon which judgments will be based need to be agreed (6b). These will include environmental indicators such as carbon emissions and particulate matter concentrations, health outcomes such as cardiovascular and pulmonary disease burdens, and intermediate indicators such as transport modal share and electricity supply. A number of models have been developed (including CRAFT, microsimulation, System Dynamics, and air pollution) to compare the potential effects of actions on environmental and health outcomes. These need to be assessed for relevance to city context and data availability and may need to be adapted to examine new exposures and outcomes, particularly those related to health (6c).

***7. Compare scenarios***

The focus of the action model is on collaborative production of knowledge that can be used to plan city actions. Our hope is that a user-friendly package of evidence can be introduced into the planning process. The package may include summaries of knowledge on the potential effects of actions based on global experience (7a), summaries of understanding of city priorities, processes, and practicalities (7b), and models that decision-makers can use to help them weigh the benefits and costs of actions (7c). The city objectives and compendium of global and local knowledge are used in discussions of possible city actions, aided by models of their potential effects.

***8. Plan together***

In this activity, city stakeholders identified in activity 2 are brought together by the research team in order to make the planning process participatory (8a). The kinds of evidence and scenario-testing that have been enabled by knowledge co-production are used in discussions with decision-makers, business leaders, non-government and community-based organisations, academics, and residents (8b). These discussions are facilitated to ensure equality of participation and contributions from critical players. Training for journalists helps them to increase the output and quality of communication about health and sustainability (8c), which engages residents with an increased level of public discussion and awareness, and the potential for engagement in participatory planning processes.

***9 and 10. Develop implementation plans and implement them***

The focus of these two stages is on the development and implementation of plans. In this activity, policymakers and decision-makers are supported to develop implementation strategies so that participatory plans have the best chance of improving city health and sustainability. This will involve the establishment of methods for year-by-year tracking of the environmental and health changes in CUSSH partner cities.

### Change model

The change model ( Figure 2) complements the action model. The action model outlines how the aims of CUSSH will be achieved, whereas the change model describes and explains the kinds of changes that we expect CUSSH should lead to, beyond the chain of outputs to meet our programme aims. The change model outlines where changes occur: in people, processes, policies, practice, and research. The change model describes the anticipated changes resulting from CUSSH in relation to its fundamental aim: to improve capacity to guide transformational health and environmental changes in cities. In developing the change model, we were drawn to research from a number of fields including systems thinking, which illustrate multi-layered outcomes, the connections between outcomes, and feedback loops between them. Although the model illustrates three broad areas of change, there is a need to appreciate that changes are non-linear. They are connected by multiple feedback loops, will occur at different levels over different periods, and are not the “end” or impact, but are likely to initiate further projects, activities and actions.

***Changes in people***

At this level, the model focuses on the changes in individuals involved in the programme (researchers, policymakers, city residents). These changes will vary with individual backgrounds. Generally, however, they will be related to individual characteristics: enhanced knowledge and understanding, improved skills, and increased capacity. They will also affect group or interpersonal relations in networks between researchers, between researchers and organisations, and between different organisations. An example of an outcome at this level is a perceived increase in the value of scientific evidence in urban decision-making.

***Changes in processes***

This level refers to changes in the processes formally or informally employed within the programme, such as processes for networking, collaboration, participation, question formation, analysis and knowledge use. The level takes a broader view of the programme in terms of its implications for ways of working, influencing institutions and norms: from the achievement of successful collaboration between diverse partners, through knowledge gathering that emphasises diversity of sources, to the achievement of meaningful participation of diverse voices and an understanding of the potential role of research in planning cities. An example of an outcome at this level is an understanding of the kinds of processes that enable transdisciplinary collaboration.

***Changes in policy, practice, and research***

This level covers organizational, institutional or disciplinary changes in practice, research, and policymaking. An example is potential interest from decision-makers in other cities in the findings and the processes themselves. The change model focuses on the anticipated changes resulting from the programme, detailing where they happen and what they are. These changes can be subtle, starting at the micro-level, but combining to seed macro-level change and the emergence of new ideas, which may lead in turn to transformative synergies ^13^. The chains of contributions and pathways that lead to change need to be examined. It is also important to consider how changes combine and fit with wider societal and environmental changes, alongside any unintended consequences that may result from the programme.

### Assumptions in the programme theory

Each component of the action and change models involves a set of assumptions. Our major assumptions, developed by a working group of representatives from the CUSSH team in finalising the programme theory, are presented in Table 5. There are also a series of higher-level assumptions:

**Table 5.** Assumptions linked to the Action Model.

Action	Assumption
1 Build relationships and consensus	Different partners desire and are able to meet and discuss their objectives, roles, and assumptions. The discussions and working together lead to a coherent vision.
2 Understand city context	A deep enough understanding of context can be developed and is sufficient to be useful in objective setting and planning. Participants derive benefits from a System Dynamics approach. City reports are granular and directional enough to be useful in the planning process. Willing and able partners can be found.
3 Synthesise city evidence synthesis	Existing policies and actions can be identified, understood and do not change too rapidly to be useful. Diagrams and simulations are developed and are then useable in the planning process. Intersectional concerns are not an insurmountable barrier to action.
4 Synthesise global evidence 5 Assess if objectives can be met	A sufficient extent of relevant global evidence has been reported in the academic literature It is possible to extract transferable knowledge from disparate global evidence. Stakeholders find global evidence intelligible and relevant to their situations.
6 Build and use models	Relevant datasets are available. Agreement is reached on indicators for each city. Models and microsimulation can generate useful data at city level.
7 Compare scenarios	Decision-makers are interested in comparing scenarios and find the comparisons credible. Plans can vary on the basis of model predictions and simulations are given weight in decision situations with multiple competing agendas.
8 Plan together	Meaningful interaction between decision-makers, researchers, and citizens is possible. Approaches to participatory planning are seen as feasible. Media output can be affected by training and decision-makers respond to it.
9 & 10 Develop and implement plans	Plans translate into actions at city level.

1. That the programme is working with the “right” decision-makers who are able to form, adopt, and implement policy.
2. That scientific evidence is an important form of information for policy and planning and that it can be certain enough to assure that its translation will lead to expected outcomes.
3. That the programme will be able to manage and integrate evidence from different sources and with different epistemologies.

### The programme theory and evaluation

The CUSSH programme theory is a pragmatic synthesis ^21^, which we hope will help us address two different types of question: one about if and how well CUSSH ‘works’ for city health and sustainability, and another about if and how well it ‘works’ as a model transdisciplinary approach. The action and change models provide the framework on which we intend to base the evaluation of CUSSH interactions with cities. Specifically, our proposal is to assemble evidence on each of the pathways.

The umbrella question is about the programme’s role and influence in bringing about transformative change in health and sustainability in cities:

1. How effective is the programme in building capacity to guide change in health and sustainability: in knowledge, awareness, skills, and tools?
2. Where and how does change occur: for people, processes, policies, practice, or research?
3. How does the programme enable the incorporation of scientific evidence, knowledge, and tools in decision and policymaking?
4. To what extent does the programme achieve participatory and transdisciplinary processes in knowledge production?
5. What lessons can be learnt for the future? What is the legacy of the programme?

The evaluation aims to answer these questions and assess the programme theory. Currently in development, the detailed evaluation framework will be "integrative", covering the processes, outcomes, and (where possible) impacts of CUSSH. It is widely recognised that “processes and outcomes cannot be neatly separated… because the process matters in and of itself and because the process and outcome are likely to be tied together” ^88^. Our evaluation framework brings together what we can learn from the processes of the transdisciplinary research programme and what difference these processes actually make. It is tied to the programme theory, as the action model describes a series of objectively verifiable outputs along with the activities or inputs that lead to them. We can examine the extent to which each action was undertaken and generate assessments of the extent to which actions achieved their goals ^89^. In the evaluation framework, the components of the action model are broken down to examinable process and output indicators. This will enable us to demonstrate how each component has been achieved, the methods that will be used to capture information, and the points at which they will be used. Indicators are also tied to specific evaluation questions; for example, were there discussions of individual interests, was stakeholder mapping done, were relevant models chosen, and were authentic and inclusive participatory public engagement events held?

The change model is broken down into outcome indicators. Alongside these indicators are a series of reflective questions about the success of the programme, tied to principles for complex, transdisciplinary, partnership projects. For example, how successful was the programme in incorporating data from diverse sources, and to what degree did decision-making use scientific research? The achievement of working relationships is less easy to assess, but is crucial and can be examined to a limited extent through reports of meetings, participant observation, and interviews with team members.

We are developing embedded, flexible, and inclusive pragmatic and systems thinking methods for evaluating this complex multilevel, multicomponent, multi-agency programme given the evolving process of transformative impacts in cities. Evaluating progress toward the overall goal of environmental and health improvement depends upon a set of agreed indicators against which such progress can be measured, and whether these indicators are applied in the cities, which is a key part of the research.

## Discussion

We have described the process of developing a programme theory for the international collaborative research of the Complex Urban Systems for Sustainability and Health project. The programme seeks to address two issues: the co-production of knowledge and its use in city policymaking, and the changes that arise from attempts to achieve transdisciplinary working. These foundational ideas underpin the programme’s aspiration: city transformation for environmental quality, sustainability, and health. Examining our approach has exposed the benefits and challenges of developing a programme theory for a complex, transdisciplinary research collaboration. A deeper appreciation of the processes has raised questions around the understanding and evaluation of such research programmes.

In this section we discuss briefly the benefits of our approach, firstly with respect to teamwork: examining team members’ preconceptions, achieving shared language amongst the team, and negotiating objectives, alongside achieving coherent action by all partners aligned with the agreed objectives. Secondly, we consider the benefits of creating a provisional framework for guiding an evaluation. Finally, we consider the challenges of our approach (practicalities, limitations, and dealing with complexity). Based on these findings we are hopeful that our example will help to overcome perceived and actual barriers to developing programme theories for complex programmes.

### Teamwork: participation and negotiation

Participatory approaches to evaluation are commonly applied in international development projects. They tend to involve a range of voices, perspectives, and knowledges in the evaluation process, sometimes including the development of community-based theories ^90, 91^. Guijt and Gaventa suggest that by “broadening involvement in identifying and analysing change, a clearer picture can be gained of what is really happening on the ground” ^92^. This ethos informed the involvement of the wider CUSSH team (including partners in different organisations) in participatory development of the programme theory.

Arriving at a programme theory that was understandable and captured the perceptions of all partners was difficult. However, developing it in a participatory way helped bring together a large and diverse partnership. Transdisciplinary initiatives are becoming important for health and development ^62, 63^, and there is an appreciation of the difficulties that arise when researchers from different disciplines work together ^1, 2^. The challenges are greater when partnership also involves political actors, civil servants, non-government organisations, and lobbying groups with different motivations, ideas about scientific evidence, and ways of working. Working together to develop a programme theory provides the space and the opportunity to appreciate and discuss these potential differences. 

We also hoped that developing the programme theory in a participatory way would contribute to a shared understanding of the programme. Shared purposes and goals do not necessarily mean that all parties have reached consensus. Instead, it is helpful to consider this process as ‘extended epistemology’, as used in action research, which sees knowledge as more valid if it is grounded in experience (experiential knowing) ^93^, expressed through stories and images (presentational knowing), understood through theories that make sense (propositional knowing), and expressed in worthwhile action (practical knowing) ^94^. Our approach enabled us to develop a clearer understanding of planned processes and anticipated outcomes from different perspectives. There is an expectation that the participatory process itself builds knowledge and skills and changes attitudes within organisations, embedding evaluative systems and practices to ensure that a culture of evaluation is sustained ^95^.

Central to both theory and practice is the cultivation of a collaborative environment: partners getting to know each other, understanding potentially different motivations, feeling safe to contribute, and settling into team roles. This takes time ^2^. The 18 rounds of discussion and review of drafts were a way to develop shared objectives and shared language. Within our example, involvement from different team members varied according to interest, workload, capacity and priorities.

Two deeper, connected issues are the possibility of conflicting interests and imbalances of power, and the hierarchy of knowledges in the process of co-production. The reality of the programme is that it is driven by academics and that different stakeholders will have different views of ‘optimal’ city actions. This is accompanied by a sense that academics are developing an ‘offer’ to policymakers and city decision-makers. This offer is transdisciplinary in that academics from different disciplines are working together with practitioners, but the knowledge products offered may favour the scientific and the peer-reviewed. In terms of power differentials, the evidence provided to policymakers privileges these types of knowledge. At the same time, however, greater power lies with city decision-makers to set the scope of what is possible and to reject the offer. What we have observed to date is a series of offers and modifications to offers that only move forward if they are seen as opportunities for city decision-makers. In a sense, the influence of the scientific orientation (however skewed) is subservient to the influence of political pragmatism and local and individual agendas. As the programme develops, our evaluation processes will encourage reflection on the different experiences of the stakeholders involved, to unpack different motivations and values that underlie such transdisciplinary projects.

### Guiding the evaluation

In some cases, a programme theory is developed by an individual project manager or evaluator. In others, it is developed in consultation with people involved in the project. There is a strong argument for complex programmes to move beyond traditional external or top-down evaluation designs to include pragmatic participatory approaches that emphasise collaborative and transformative processes in knowledge production.

All programme theories are provisional ^80^, and evaluation should test the theory itself. This means that evaluation is operating at two different levels: a type of process evaluation in which we assess whether certain things happen (were the inputs sufficient, was the programme managed well enough?) and whether the theory holds (were certain stages necessary, did the theory fit what happened?). The CUSSH theory points to outputs and processes that can be assessed by a combination of qualitative and quantitative methods. This kind of evaluation will help partners to better understand the objectives, challenges, opportunities, and strategies needed to improve capacity for transformative changes in city health and sustainability. Framing the theory and evaluating the programme also helped to draw out areas of uncertainty; for example, what is meant by terms such as stakeholder, intervention, and governance.

### Practical challenges

The subgroup that worked on the programme theory had 25 members and the collaboration as a whole about 60 members. Despite in-person and digital conferencing and teamworking applications, subgroup meetings were rarely attended by more than six members at a time. All the members of the theory subgroup were researchers and almost all were from high-income countries, which raises the question of how representative the subgroup was of the whole. We had to judge appropriate points in the development process at which to share iterations of the programme theory, and time and agenda pressures made it hard for partners to get a firm enough grasp to contribute. To enable full participation in the development of a programme theory there is a need to ensure a variety of means to contribute (workshops, reviews of documents, meetings, working groups), and that members have the tools, capacity and confidence to engage with them. We stress that the different ways of knowing and different expertise all have a role to play in theory development.

### Limitations

Throughout the process of developing the programme theory, there was a tension between generality and specificity. For example, the city of Kisumu identified the problem of solid waste management as important. Although this became an ‘agreed specific city objective’ on which we are working, those involved in the Kisumu work noted that the general model feels abstract and does not extend to this level of detail. We envisage that the programme theory will be used to generate specific versions that address individual cities and concerns. What is important is to repeatedly assess whether what happens locally fits the more general pattern.

A second debate was whether the theory was ‘about’ achieving urban sustainability and health or ‘about’ the co-production of knowledge and transdisciplinarity. Both are aspirations and both are difficult to achieve. Our decision to address them in separate models within one pragmatic theory was a way to clarify the dimensions in which the programme will be evaluated. Although it took two years of feedback, discussion, and revision to reach the current version, we have not yet seen or evidenced some of the later part of the model, notably the process of implementation. Since our focus is on the use of scientific knowledge to improve city health and sustainability, the way that this is enacted is necessarily through a process of planning. The fidelity with which plans are implemented, the likelihood that they will be modified by street-level bureaucracy ^96^, and unsanctioned city change will affect the likelihood of plans becoming reality.

Implementation also depends on the likely weights of other concerns—financial, electoral, ideological—in decision-making ^49^. The use of health-related evidence in urban policy can be swamped by other guidance and legislation ^41^. Our programme responds particularly to ideas about what is needed for cities to maximize co-benefits for environment and health. The aspiration is to apply systems thinking. Doing so, however, implies identification of people and organisations working across cities, and policies and their relative power and interaction, combining them with the lived experience of residents in participatory decision-making processes. Encouraging deeper dialogue through public awareness activities and interest groups is important ^52^, but a substantial challenge when public engagement in policymaking in many cities extends to a process of consultation on plans that are already fairly advanced.

### Dealing with complexity

The theory describes a complex programme. Representing it in a two-dimensional figure might make it look unidirectional and overly linear. Implicit in the idea of complexity is unpredictability, emergence, and non-linearity, all of which are embedded in our programme theory. It is true that feedback loops are ubiquitous and that many of the activities take place simultaneously. Nevertheless, we think that there is a degree of linearity because steps are conditional on preceding steps. We cannot achieve anything without working relationships, and we cannot develop models without understanding global and local precedents.

We also found representing time within the theory challenging. In the face of repeated human resource changes—in research institutions, municipalities, and private organisations—election exigencies, and the coronavirus disease 2019 (COVID-19) pandemic, the idea that a programme could move steadily from one state to another seems simplistic. If the bedrock of collaboration is working relationships, we have to accept that they will almost certainly need to be cemented repeatedly from scratch over the life of a project. For example, as a new mayor arrives, people with existing relationships leave, or activities are suspended for six months because of other pressing issues.

## Conclusion

We argue that developing a programme theory is a key part of the process of understanding how successful programmes can be constructed and how change is actually brought about. It was an important step in bringing together a diverse team to articulate a vision of a complex project with diverse partners in different countries and with a range of roles and backgrounds. It helped in team building and identifying how many—often simultaneous—activities might come together. It provided a preliminary framework for understanding the programme processes that could be evaluated and for returning to assess whether propositions held.

Partners in multi- and transdisciplinary programmes should begin discussing theory as early as possible, not least because this provides a means of surfacing assumptions and views on important mechanisms and is a way of strengthening teams. Achieving consensus from many individuals and organisations requires an extended period of iteration, response to suggestions, and redrafting. Although consensus might not be possible, space needs to be created to discuss questions about the power dynamics that underlie the processes of co-production, transparency in decision-making, different forms of evidence being ‘optimal’, and varying conceptions of what ‘success’ looks like.

Examining our approach has exposed the benefits and challenges of developing a programme theory for a complex, transdisciplinary research collaboration. Despite the challenges and limitations, opening the “black box” is necessary for understanding how successful programmes can be constructed and how change might be brought about.

## Data availability

### Underlying data

Open Science Framework: CUSSH programme theory. https://doi.org/10.17605/OSF.IO/6EB9S ^86^

This project contains the following underlying data:

- Post_its.zip (zip folder with images of post-it notes)
- Toc_1.pptx – toc_17.pptx (archives of various versions of the programme theory)
- CUSSH_ToC_development.docx (document explaining the changes between versions)
- CUSSH_theory_archive.pdf (CUSSH Theory of change)

Data are available under the terms of the Creative Commons Attribution 4.0 International license (CC-BY 4.0).
