# Supplementary material for: Developing a programme theory for a transdisciplinary research collaboration: Complex Urban Systems for Sustainability and Health
Source: Wellcome Open Res. 2021 Jul 14;6:35. Originally published 2021 Feb 19. [Version 2] doi: 10.12688/wellcomeopenres.16542.2 (PMC8156501; doi:10.12688/wellcomeopenres.16542.2)
Supplement: Peer review history

# Peer review: Reviewer response for version 1

**DOI:** 10.21956/wellcomeopenres.18228.r43715

**Published:** 2021-05-26

**Authors:** Timothy H. Ciesielski

**Affiliations:** 1 Department of Population and Quantitative Health Sciences, Case Western Reserve University School of Medicine, Cleveland, OH, USA; 2 Ronin Institute, Montclair, NJ, USA

The authors of this article describe the development of a program theory to guide their massive transdisciplinary project (13 institutions in 6 cities across 3 continents). Their overall aim is to bring researchers, stakeholders, and decision makers together to improve environmental quality, sustainability, and human health in cities. In short, they want to know if knowledge and action can be co-produced to advance human health and sustainability in cities, and they also want to know if transdisciplinary approaches can make this happen. These are noble and challenging goals.  The description of the current state of this project is a very useful addition to the literature, even as (and perhaps because), many questions remain.  

 For context: My review flows from my perspective as an academic researcher with a background in epidemiology and evidence integration across academic disciplines. More recently I have engaged in projects that include community voices. I have a few comments and questions:

1. A program theory does seem useful, but could empirical observations also be feasible and important? In other words, are there empirical transdisciplinary observations of cities that improved or worsened with regard to human health and sustainability? These observations may yield idiosyncratic patterns or locally relevant anecdotes, but in aggregate they might reveal evidence for the existence of universal fundamental drivers of these two outcomes (e.g., corruption, government type, common local industries, human population growth, or economic models that myopically support human population growth).  
2. Are all stakeholders acting in good faith? Can this be known? Can this be handled?
  Transdisciplinary” is not always defined in the exact same way by all researchers, but in any case, this project is “transdisciplinary writ large”. In “transdisciplinary writ small” the participants are from academic fields, and these fields typically share a common process and goal: the use of evidence to describe and explain our world. However, once stakeholders and decision makers are folded into the mix, several alternative prime directives may appear among the participants. Evidence for learning may take a back seat to the maintenance of financial or political power for some people. This is not to say that researchers are without bias or conflicts or drama, but it is to note that they share a vague goal and a common standard of semi-verifiable conduct. How can co-learning and co-production be conducted when some participants have conflicting goals? Can all of stakeholders serve as good faith participants? Is obstruction of the transdisciplinary process a near-term success for some of those that benefit from the status quo? How could you identify these actors? If you could identify them, how could you deal with them ethically?
3. This paper provides an extensive collection of details, stories, and processes. These details are needed and they raise questions for future work. Having said this, could providing a distilled description of the approach further increase the utility of the manuscript and the number of readers?  
4. The assumptions are key, and it is excellent that they are clearly enumerated, but I wonder if they also present latent opportunities. Evidence can only be considered when it exists. What about ideas and interventions for which there is yet no evidence? If researchers could explicitly highlight emerging ideas that need evidence, this might reveal latent synergy. Policy makers may be able to provide government funding for evidence collection, and researchers need that funding in order to fill the evidence voids (and keep their jobs). This approach is not without peril but it may be worth investigating.

Is the work clearly and accurately presented and does it cite the current literature?

Yes

If applicable, is the statistical analysis and its interpretation appropriate?

Not applicable

Are all the source data underlying the results available to ensure full reproducibility?

No source data required

Is the study design appropriate and is the work technically sound?

Yes

Are the conclusions drawn adequately supported by the results?

Yes

Are sufficient details of methods and analysis provided to allow replication by others?

Yes

Reviewer Expertise:

Addressing complex health problems with epidemiology and transdisciplinary evidence integration.

I confirm that I have read this submission and believe that I have an appropriate level of expertise to confirm that it is of an acceptable scientific standard.

# Peer review: Reviewer response for version 1

**DOI:** 10.21956/wellcomeopenres.18228.r42826

**Published:** 2021-03-19

**Authors:** Marcela Brugnach

**Affiliations:** 1 BC3 Basque Center for Climate Change, Bilbao, Spain

In this research article, the authors propose a programme theory for transdisciplinary research collaboration to improve the capacity to guide transformational health and environmental changes in complex urban settings. An interesting project, with equally interesting results. 

 I have the following questions/comments: 

1. My main concern is over the use of the concepts of transdisciplinary and the co-production of knowledge adopted in CUSSH. While it is asserted that different ways of knowing and different forms of expertise all have a role to play in theory development, the roles ascribed are far from equal. The emphasis is placed on scientific knowledge and expertise being the legitimate knowledge sources. This raises fundamental questions regarding what type of co-production is done in practice, and in what role and with what purpose non-expert actors are involved, how local knowledge is included in other words. Is CUSSH really addressing transdisciplinarity, or bringing interdisciplinarity into practice?
2. Optimal and transdisciplinary: you claim that "The programme’s aim is to enable city decision-makers to select and implement optimal actions for both sustainability and health", but an optimal action in a transdisciplinary context, may in fact be a less than optimal action in science. I think what is meant by optimal, is a point that deserves further clarification. 
3. Placing CUSSH in practice. How do you align the temporal scale of the transformation or action sought after on the ground with the project? What happens after this project is completed, is there any follow up? If the goal of the programme is to stimulate transformation, how is continuity guaranteed? What happens with the relationships established among actors post-project?
4. What do you mean by "time-poor" policymakers?
5. I think it would be very useful for the reader to expand on the challenges faced when developing and applying such programme theory; e.g., How do you address conflicting interests and power differentials among actors? What difficulties arise when researchers from different disciplines work with each other and with partners from other sectors? etc.

Is the work clearly and accurately presented and does it cite the current literature?

Yes

If applicable, is the statistical analysis and its interpretation appropriate?

Not applicable

Are all the source data underlying the results available to ensure full reproducibility?

Yes

Is the study design appropriate and is the work technically sound?

Yes

Are the conclusions drawn adequately supported by the results?

Yes

Are sufficient details of methods and analysis provided to allow replication by others?

Yes

Reviewer Expertise:

Collective decision making under uncertainty in natural resource governance. Knowledge coproduction. Participatory processes.

I confirm that I have read this submission and believe that I have an appropriate level of expertise to confirm that it is of an acceptable scientific standard.
